# Association Between HDL Cholesterol and QTc Interval: A Population-Based Epidemiological Study

**DOI:** 10.3390/jcm8101527

**Published:** 2019-09-23

**Authors:** Rosaria Del Giorno, Sofia Gabutti, Chiara Troiani, Kevyn Stefanelli, Raffaele Falciano, Elisa Graziano, Tommaso Rochat Negro, Luca Gabutti

**Affiliations:** 1 Department of Internal Medicine and Nephrology, Clinical Research Unit, Regional Hospital of Bellinzona and Valli, Ente Ospedaliero Cantonale, 6500 Bellinzona, Switzerland; 2 Department of Social Sciences and Economics; Sapienza University of Rome, 00185 Rome, Italy; 3 Institute of Biomedicine, University of Southern Switzerland, 6900 Lugano, Switzerland

**Keywords:** HDL cholesterol, ECG, QTc interval, epidemiological study, population-based study, QTc prolongation risk

## Abstract

Previous experimental studies showed that increasing high-density lipoprotein cholesterol (HDL) cholesterol shortens cardiac ventricular repolarization and the QT interval corrected for heart rate (QTc). However, little is known about the epidemiological relationship between HDL and QTc. The potential antiarrhythmic effect of HDL cholesterol remains a speculative hypothesis. In this cross-sectional population based study in adults living in the Italian-speaking part of Switzerland, we aimed to explore the association between HDL cholesterol and the QTc interval in the general population. A total of 1202 subjects were screened. electrocardiogram (ECG) recordings, measurements of lipid parameters and other laboratory tests were performed. QTc was corrected using Bazett’s (QTc_Baz_) and Framingham (QTc_Fram_) formulas. HDL was categorized according to percentile distributions: <25th (HDL-1; ≤1.39 mmol/L); 25th–<50th (HDL-2; 1.40–1.69 mmol/L); 50th–<75th (HDL-3; 1.69–1.99 mmol/L); and ≥75th (HDL-4; ≥2.0 mmol/L). After exclusion procedures, data of 1085 subjects were analyzed. Compared with the HDL reference group (HDL-1), HDL-2 and HDL-3 were associated with a reduction of QTc_Baz_ and QTc_Fram_ duration in crude (HDL-2, QTc_Baz_/QTc_Fram_: β-11.306/–10.186, SE 4.625/4.016; *p* = 0.016/0.012; HDL-3, β-12.347/–12.048, SE 4.875/4.233, *p* = 0.012/<0.001) and adjusted (HDL-2: β-11.697/–10.908, SE 4.333/4.151, *p* < 0.001/0.010; HDL-3 β-11.786/–11.002, SE 4.719/4.521, *p* = 0.014/0.016) linear regression models in women. In adjusted logistic regression models higher HDL, were also associated with lower risk of prolonged QTc_Baz_/QTc_Fram_ (HDL-2: OR 0.16/0.17, CI 0.03–0.83/0.47–0.65; HDL-3: OR 0.10/0.14, CI 0.10–0.64/0.03–0.63) in women. Restricted cubic spline analysis confirmed a non linear association (*p* < 0.001). The present findings indicate an epidemiological association between HDL cholesterol and QTc duration. To draw firm conclusions, further investigations in other populations and with a prospective cohort design are needed.

## 1. Introduction

In recent years, an intriguing antiarrhythmic effect of high-density lipoprotein cholesterol (HDL) was postulated. Studies in animal models found a direct association between post-infarction plasma HDL levels and ischemia-reperfusion related ventricular arrhythmias (VA), with high plasma HDL levels exerting a protective role [1,2]. Furthermore, low HDL cholesterol levels are linked with increased risk of sudden cardiac death [3,4,5], and several reports have postulated a correlation between plasma HDL concentrations and the onset of fatal arrhythmias [6,7]. Last but not least, lower circulating HDL levels in patients with paroxysmal atrial fibrillation were found [8,9,10].

Ventricular tachyarrhythmias often precede cardiovascular death and the prolongation of the ventricular cardiac repolarization time, which often underlies these rhythm disturbances, is one of the predictors of sudden cardiac death [11,12,13].

A previous experimental study revealed that increasing HDL cholesterol shortens cardiac ventricular repolarization in isolated cardiomyocytes and the QT interval corrected for heart rate (QTc) in healthy humans [14].

The electrocardiographic QT interval mainly reflects cardiac ventricular repolarization, and prolongation of the heart rate-corrected QT interval (QTc) has long been recognized as a marker of sudden cardiac death, cardiovascular death, and all-cause mortality [15,16] both in patients with prevalent coronary heart disease and in middle-aged and older adults without prior cardiovascular disease (CVD) [17].

Despite the experimental evidence indicating that increasing levels of HDL could exert a direct effect on the cardiac repolarization, specifically shortening the QT interval, little is known about the epidemiological relationship between HDL and QTc in humans, and whether HDL cholesterol concentrations are associated with the risk of QTc prolongation. 

Indeed, the association between HDL concentrations and QTc interval has been investigated only in one study on 440 primary hypercholesterolemic patients, which failed to show any association between HDL cholesterol levels and the QTc interval [18].

To date, several questions about the association between QTc and HDL remain open: Does HDL exhibit the QT-shortening effect also in vivo and in non-ischemic myocardium? What is the magnitude of this effect in the general population? Which type of relationship between HDL levels and prolongation of ventricular repolarization exist (e.g., linear, dose-response)?

In this study we sought to explore the possible association between HDL concentrations and the QTc interval in the general population, hypothesizing a non-linear association between HDL levels and the risk of QTc interval prolongation.

This investigation could lead to a better understanding of underrecognized pleiotropic effects of HDL cholesterol on the heart.

## 2. Methods

### 2.1. Study Population

The Ticino epidemiological stiffness (TEST) study was a population-based, observational study of adults aged ≥18 years, were residents in Ticino, and part of the Italian-speaking part of Switzerland. The baseline examination took place between June 2017 and July 2018. The study was designed to investigate the impact of several cardiovascular risk factors on arterial stiffness. Participants were recruited by mail using a simple random sampling method, on the basis of a list provided by the Swiss Federal Statistical Department in which the resident population is organized by the district of residence, gender, and age group. In total, 1400 residents were invited of whom 1202 (86%) participated by completing the study protocol. Ticino is a small Swiss region of 322,000 inhabitants, divided in four districts (Bellinzona and Valleys, Lugano, Locarno and Valleys, and Mendrisio) among which differences in habits and lifestyle can be found. For this reason, the population recruitment was performed taking into account the sub-regional residency, and a homogeneous-district recruitment, based also on the district-area of residency. A novel aspect of the TEST study was the creation of a regional Swiss database to address geographic differences in arterial ageing and cardiovascular risk factors. The study was carried out in accordance with the Helsinki Declaration and was approved by the local Swiss ethics committee. All participants provided informed written consent. Data and analyses are presented in accordance with the Strengthening the Reporting of Observational Studies in Epidemiology (STROBE) [19]. At the end of the study every participant and their physician, as requested by the participant, received information by mail on all medical and laboratory tests performed during the study.

### 2.2. Measurements

#### 2.2.1. Clinical Evaluation 

The study participants underwent a detailed medical examination and a standardized interview, in which socio-demographic, behavioral, and nutritional habits were collected. Personal and family medical history and pharmacological anamnesis were also performed. Demographic and clinical characteristics of subjects included: Age, educational, and civil status. A detailed personal CVD anamnesis for previous coronary heart disease (CHD), cerebrovascular disease (CeVD), and peripheral artery disease (PAD) was performed. All traditional cardiovascular risk factors were recorded, including: Hypertension, type 2 diabetes, hypercholesterolemia/hypertriglyceridemia, smoking status, and kidney function. History of previous episodes of heart arrhythmias was also registered. All the above mentioned information on CVD anamnesis was collected also for nucleus family members (parents/brothers/sons). Pharmaceutical anamnesis, including current and previous medication, was also performed. All types of generic drug names including the commercial one, the dose, and treatment duration were registered. Physical examination included height (cm), weight (kg), waist circumference (cm), hip circumference (cm), body mass index (BMI; Kg/m^2^), and neck circumference (cm). Blood pressure (BP) was measured during the first examination using a validated automatic oscillometric device and following standardized procedures [20] (Dinamap model Pro 100 automated oscillometric sphygmomanometer, Critikon, Tampa, Florida). The average of the last two of three consecutive BP measurements was considered as the reference value. Thereafter, all patients underwent a 24-h ambulatory BP monitoring (ABPM) using a validated, automated noninvasive oscillometric device (Mobil-O-Graph, I.E.M. GmbH, Stolberg, Germany) [21] programmed to register BP every 30 min during daytime and every hour during nighttime respectively. Appropriate cuff sizes were used. The ABPM recordings were performed on working days, and the patients were instructed to maintain their usual activities. Daytime and nighttime periods were pre-defined. Arterial stiffness was evaluated by pulse wave velocity (PWV) measurements during the initial visit at three different artery sites using two different techniques. The gold standard technique, based on a non-invasive applanation tonometric device (SphygmoCor Cardiovascular Management System, version 8.2, AtCor Medical, Australia) was used first [22]. With this technique the carotido-femoral PWV was assessed and the radial artery PWV was estimated by pulse wave analysis (PWA). The second PWV analysis technique consisted of an oscillometric device (Mobil-o-Graph,) allowing simultaneous recordings of blood pressure and PWA during cuff occlusion of the brachial artery [21]. The participants were instructed to abstain from alcohol for 12 h and from caffeine and tobacco for four hours. A body composition analysis was also performed. Impedance measurements were taken after 10 min of rest with a BIA impedance analyzer (BIA 101, Akern Bioresearch, Florence, Italy).

#### 2.2.2. Laboratory Measurements

Blood samples were drawn from every participant from the cubital vein or one of its branches in the supine position and prepared for immediate analysis or stored at −80 °C for delayed analysis. Lipid, renal, glycemic, and electrolytic profiles were determined.

HDL cholesterol, LDL cholesterol, triglycerides, and total cholesterol levels were measured from blood samples obtained after a 12 h fast using standard assays (Roche Diagnostics, Mannheim, Germany). Serum creatinine, uric acid, fasting plasma glucose, glycated hemoglobin (Hba1C), and total serum magnesium were assessed using an automatic biochemistry analyzer (Roche Cobas 8000 modular analyzer Series C701, Mannheim, Germany).

Serum cystatin C was determined using the Roche Cobas 8000 modular analyzer series (Roche, Inc., Mannheim, Germany) with a particle-enhanced immunonephelometric assay (Siemens).

Renal function was then determined by cystatin C-derived eGFR (eGFRcys) according to the following formula: eGFRcys = 76.7 × cystatin C [23]. Ionized calcium, pH, and ionized magnesium were measured using an ionometer (Microlyte 6 Analyzer, Kone Instruments, Espoo, Finland).

Furthermore participants were asked to perform a 24 h urine collection, in which several analyses were performed, including sodium and potassium (measured using a Roche Hitachi; an indirect ion-selective electrode). Urinary albumin concentration and creatinine were determined using a Behring Nephelometer (Siemens BN albumin; Siemens Healthcare, Marburg, Germany) and a Hitachi 717 device (Roche Diagnostics), respectively.

#### 2.2.3. Electrocardiogram Analysis 

Each participant underwent an electrocardiogram (ECG) as a part of the visit examination. A standard 12-lead resting ECG (25 mm/s paper speed, 10 mm/mV amplitude, and 250 Hz sampling rate) was recorded using a MAC 5500 machine (MAC 5500/5500 HD of GE Healthcare with automated analysis by the GE Marquette 12SL ECG Analysis Program, Milwaukee, WI, USA). All ECGs were initially inspected for technical errors and quality, repeating ECGs with excessive noise potentially interfering with analysis. For each ECG, the following parameters were recorded: Heart rate (HR), RR interval, PR interval, QRS interval, and QT interval. All QT intervals were also manually calculated by four physicians using a mean of three consecutive beats derived from either lead II or V5. The end of the T wave was determined by the tangent method, and the U waves were not incorporated if they were separate from the T wave [24]. QT intervals were corrected for HR using two formulas: Bazett’s formula, (QTc: QT/√(60/heart rate)), where QTc_Baz_ is the QTc calculated using Bazett’s formula and the Framingham linear regression formula: (QTc_Fram_ = QT + 154 (1–60/heart rate)), where QTc_Fram_ is the QTc calculated using the Framingham formula, following the recommendations of the American Heart Association, American College of Cardiology, and Heart Rhythm Society for the Standardization of ECG [24]. ECGs were also assessed manually for the presence of changes that could affect the QT interval: Left bundle branch block (LLBB), right bundle branch block (RBBB), ventricular pacing, atrial fibrillation, atrial flutter, other supraventricular tachycardias, ST-T wave changes of ischemic origin, and left ventricular hypertrophy by voltage criteria. Questionable abnormal ECGs were reviewed by a cardiologist.

### 2.3. Statistical Analysis

Continuous variables were expressed as median (interquartile range) and categorical variables as a percentage. Association between HDL levels and QTc interval was examined using categories of HDL cholesterol. 

According to the HDL distribution in the study population, four HDL cholesterol percentile categories were defined, as follows: <25th; (HDL-1); 25th to <50th; (HDL-2); 50th to <75th (HDL-3); and 75th and above percentiles (HDL-4).

HDL categories based on percentiles corresponded to the following values: ≤25th percentile, HDL ≤1.39 mmol/L (≤54 mg/dL); 25th to <50th HDL 1.40 to 1.69 mmol/L (54–65 mg/dL); 50th to <75th HDL 1.69-1.99 (65–77 mg/dL); and 75th and above percentiles HDL ≥ 2.0 mmol/L (≥77 mg/dL).

In order to investigate the relative contribution of HDL categories on QTc interval duration, linear regression models were performed with the HDL category ≤25th percentile (HDL-1) considered as the reference group. All analyses were separately performed in men and women, for both QTc_Baz_ and QTc_Fram_. The β-coefficient and standard error (SE) relative to HDL categories were determined. To explore the association between HDL values of each category and the risk of QT prolongation, logistic regression models were also performed. Associations were expressed as odds ratios (OR) with 95% confidence intervals (CI). QTc prolongation was defined according to the European Society of Cardiology Guidelines: >450 ms in men, >470 ms in women for the QTc_Baz_, and considering the 90th percentile for the QTc_Fram_ (QTcFram 445 ms in women and QTcFram 438 ms in men) [24]. Both linear and logistic regression models were used: Model 1 unadjusted; and model 2 adjusted for age, diabetes, hypertension, family history of CVD, BMI, 24h-urinary-sodium excretion, 24h-urinary potassium excretion, creatinine, systolic blood pressure, diastolic blood pressure, magnesium, ionized calcium, heart rate, QRS duration, cystatin, pulse wave velocity, pulse pressure, glycemia, albuminuria, waist/hip circumference, and statin therapy. 

All covariates were included in the adjusted models to examine potential confounding effects. All traditional cardiovascular risk factors and all variables potentially affecting the QTc interval were selected. Each adjusted logistic regression model was examined separately for multicollinearity using the variance inflation factor (VIF) statistic; a VIF >4.0 being an indicator of multicollinearity. We decided not to impute the missing data after having verified that we were in the case of missing completely at random data. In order to verify the latter, we performed a regression analysis using Y as our objective variable (QTc) and X as a bunch of variables useful to determine differences between patients, like age, sex, and the number of laboratory tests. The data analysis revealed a negligible amount of missing data (two values for QTc_Fram_ and two values for QTc_Baz_), which corresponded to a missing/available data ratio lower than 0.4%. Considering that, it is not suggested to perform a multiple imputation analysis for missing data for values lower than 5% [25], we decided to carry out a complete case analysis. In addition, a sub-analysis of our small sample of subjects with missing data revealed, in the absence of classical cardiovascular risk factors, a ratio females/males of 1 and a wide age range (29–60 years).

To describe the functional relationship between HDL cholesterol and the risk of QTc interval prolongation and in order to explore the linearity of the relationship, restricted cubic regression spline analyses were performed with three knots located at the 25th, 50th, and 75th percentiles. A *p*-value for non-linearity was calculated by testing against the null hypothesis that the coefficient of the second spline transformation was equal to zero.

The use of restricted quadratic splines enabled us to model in a more realistic way the relationship between QTc and HDL, allowing us to overcome the variations within categories of HDL investigated in the multivariable logistic regression and treating HDL as a continuous variable, to achieve the desired flexibility to test the linearity of the relationship.

Statistical analyses were performed using SPSS (version 18.0, Chicago, IL, USA), and R version 3.2 software (The R Project for Statistical Computing, Vienna, Austria). All variables were considered statistically significant when the *p*-value was less than 0.05.

## 3. Results

**Figure 1:**
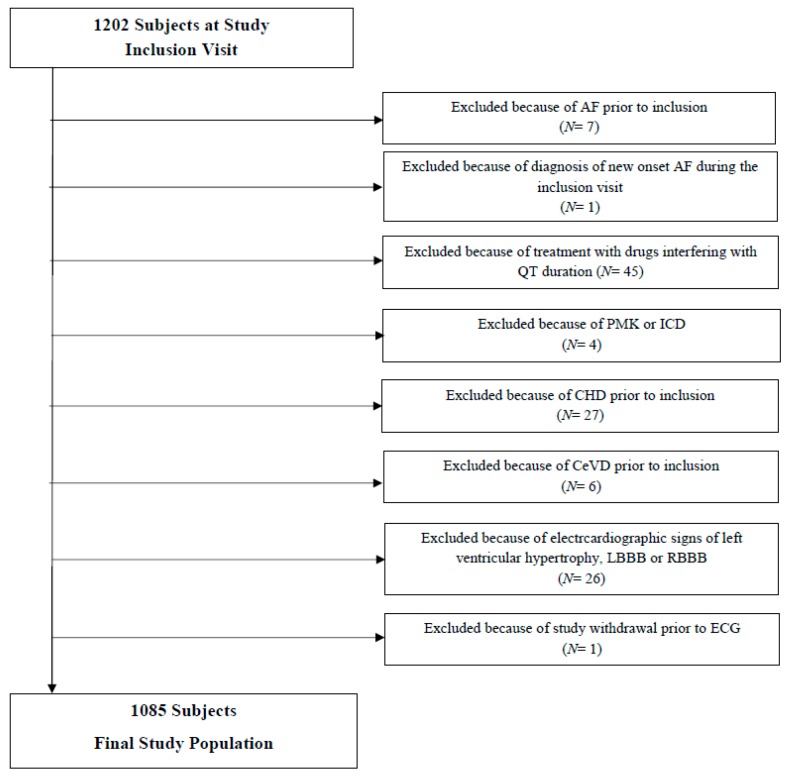
Flowchart showing the population selection procedure.

A total of 1085 participants were included in the present analyses. Subjects with missing data, ventricular pacemakers or ICDs, established coronary artery disease, ECG abnormalities affecting QT, QTc-prolonging, or anti arrhythmic drugs use were excluded. The results of the exclusion process, summarizing exclusion reasons is shown in Figure 1. The median age (IQR) of the study population was 51 years (42–59); 57.1% were women.

Table 1 shows the baseline characteristics of all individuals, as well as laboratory findings, ECG parameters and ambulatory blood pressure monitoring parameters.

The median (Q1–Q3) HDL level was 1.6 (1.3–1.9) mmol/L. Based on HDL percentiles, subjects were distributed in categories as follows: HDL-1 31%, HDL-2 28%, HDL-3 21%, and HDL-4 19%. The median (Q1–Q3) of QTc for QTc_Baz_ and QTc_Fram_ were respectively 427 (413–441) and 420 (408–432) msec.

Multiple linear regression models were fitted for men and women separately to estimate the effects of the variables of interest on Qtc.

Table 2 shows the unadjusted and adjusted linear associations between HDL levels and QTc_Baz_ and QTc_Fram_ by sex. Significant associations were found in women; in whom HDL-2 and HDL-3 were associated with a reduction of QTc_Baz_ and QTc_Fram_ in both models. In HDL-2, β estimates for QTc_Baz_ and QTc_Fram_ in unadjusted models were respectively: −11.306 and −10.186, (*p* = 0.016 and =0.012); whereas for HDL-3 results were as follows: β −12.347 and –12.048, (*p* = 0.012 and <0.001). The associations persisted in fully adjusted models, where HDL-2 and HDL-3 in women were significantly independently associated with a reduction of both QTc_Baz_ and QTc_Fram_. In men HDL-2 and HDL-3 showed a similar, even if not significant, reduction of both QTc_Baz_ and QTc_Fram_, in unadjusted models (see Table 2 and Supplementary Table S1 for further details).

Using logistic regression models, HDL-2 and HDL-3, had a considerable significant association with a reduced risk of prolonged QTc in women in both the crude and adjusted model, but not in men. 

In women HDL-2 was associated with a significant reduction of the risk of a prolonged QT_Baz_ and QT_Fram_ in the crude model, respectively: OR 0.27, CI 0.10–0.80, *p*-value 0.018 and OR 0.37, CI 0.12–1.06, *p*-value 0.064. The reduced risk of a QTc prolongation was confirmed for the HDL-2 in the adjusted model, QTc_Baz_/QTc_Fram_ OR 0.17, CI 0.03–0.83; *p*-value 0.02, and OR 0.17, CI 0.05–0.65, *p*-value < 0.001.

HDL-3 was also associated with a significant reduction of the risk of a prolonged QTc_Baz_ and QTc_Fram_ in the crude model in women, respectively: 0.21, CI 0.06–0.72; *p*-value 0.013 and OR 0.12, CI 0.02–0.58, *p*-value < 0.001. 

**Figure 2:**
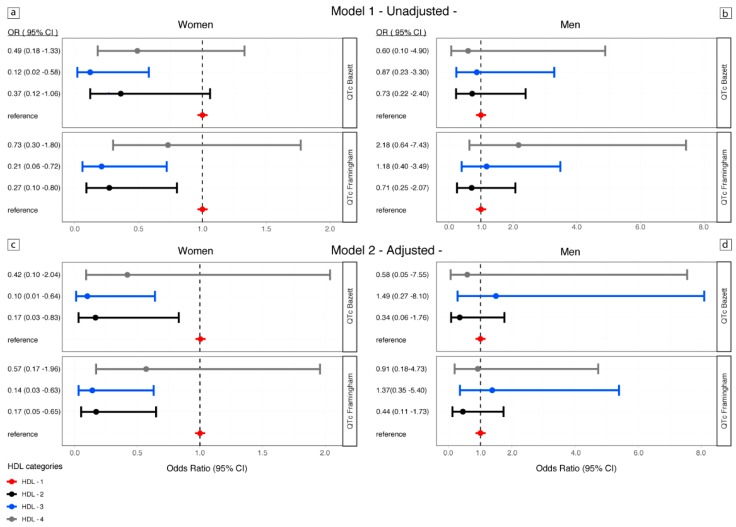
Risk of QTc prolongation (QTc_Fram_ and QTc_Bazz_), by HDL categories in men and women. Analyses were performed separately for women and men and for both QTc_Baz_ and QTc_Fram_. The risk was computed for unadjusted (top of the figure, panels a and b), and for adjusted models (bottom, panels c and d), according to HDL categories based on percentile distributions: reference groups, red line, <25th percentile (HDL-1, ≤1.39 mmol/L); dark line 25th–<50th percentile (HDL-2, 1.69–1.99 mmol/L); blue line 50th–<75th percentile (HDL-3, 1.69–1.99 mmol/L), and gray line ≥75th percentiles (HDL-4, ≥2.0 mmol/L). Odds ratios and 95% confidence intervals were reported for all models.

The significant association in risk reduction of a prolonged QTc_Baz_ and QTc_Fram_ was confirmed in fully adjusted models for HDL-3: OR 0.10, CI 0.01–0.64, *p*-value 0.01 and OR 0.14, CI 0.03–0.63, *p*-value 0.010 (Figure 2). Overall in the logistic regression analyses, where HDL cholesterol was categorized in percentiles, we found that in females, HDL-cholesterol higher percentiles (HDL-2 and HDL-3) were associated with reduced risk of QTc_Fram_ and QTc_Baz_ prolongation compared with the lowest percentile (HDL-1). The highest percentile of HDL (HDL-4) was nevertheless not significantly associated with QTc prolongation in women, not enabling us to properly define the pattern of the relationship.

**Figure 3:**
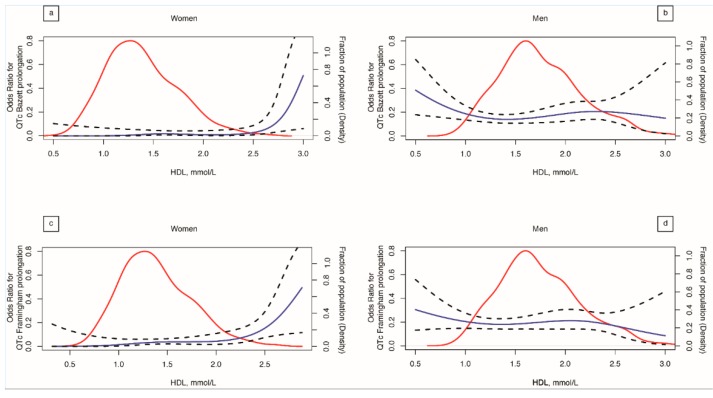
HDL cholesterol and risk of QTc prolongation. HDL cholesterol on a continuous scale and risk of QTc prolongation in the Ticino epidemiological stiffness (TEST) study. Analyses were performed using restricted cubic splines with 3 degrees of freedom. In the top of the figure (panels **a** and **b**) was the risk of QTc_Bazz_ prolongation for women and men, in the bottom of the figure was the risk of QTc_Fram_ prolongation for women and men (panels **c** and **d**). The red continuous line indicates the fraction of the population with the indicated HDL-cholesterol value (density). Dashed dark lines indicate pointwise 95% confidence intervals.

A significant deviation from linearity for women for both QTc_Baz_ and QTc_Fram_ (*p* < 0.001) was found. The restricted cubic spline analysis suggests in fact that the relationship between HDL and QTc prolongation is not linear in women and exhibits a J-shaped association (Figure 3). This relationship indicates a lower risk of QTc prolongation in the middle spectrum of HDL values and a higher risk for very high HDL levels (more than 2.25 mmol/L for QTc_Fram_ and more than 2.50 mmol/L for QTc_Baz_).

Results shown in Table 2 refer to linear regression models produced using a categorical independent variable, which expresses the expected QTc variation according to four predetermined HDL categories (HDL-1, HDL-2, HDL-3, and HDL-4). In the model, the intercept is represented by the HDL category with the lowest value (HDL-1). In Figure 3 on the contrary, a model using QTc as the independent continuous variable, and as a dependent variable the binary risk of QTc prolongation was used. The second analysis was performed to explore further the relationship between the risk of QTc prolongation and HDL, overcoming the limits set by the HDL categorization related to the linear regression. The resulting trend found was similar even if the shape of the two curves did not exactly match in both models a J-shaped relationship.

In men a significant deviation from the linearity was confirmed only with QTc_Baz_ (*p* = 0.033).

## 4. Discussion

In this study we found an association between HDL cholesterol levels and the duration of ventricular repolarization, estimated with the QTc interval, in women.

To our knowledge this data provided the first evidence that HDL cholesterol was associated with QTc duration in the general population, suggesting that HDL might play an antiarrhythmic role in humans. 

Previously, only one study was aimed at exploring, at the population level, the potential association between HDL and QTc, and failed in finding any association [18]. To note, the association was explored in a population of dyslipidemic patients with a limited sample size, and considering an incomprehensive number of confounding factors potentially modulating QTc.

Conversely, our study was based on an unselected larger population and a detailed exclusion process was performed. All factors affecting electrocardiographic QTc duration or QTc interpretation were taken into account or considered in the exclusion algorithm; this resulted in a population potentially free from confounding factors.

Although the present study was not designed to evaluate the biological phenomenon linking QTc interval duration and HDL, our data raised several interesting questions. 

To our knowledge, there is no definitive evidence of a direct causal effect of HDL on ventricular action potential duration. Nevertheless, it was recently shown that the lipid composition of the cellular membrane modulates cardiac electrophysiology and arrhythmogenesis in the absence of structural abnormalities [26,27]. It was in particular demonstrated that a variety of ion channels, including those that influence action potential characteristics, determining the QT duration, could be regulated by changes in the level of membrane cholesterol [28,29]. Moreover, experimental findings showed that dyscholesterolemia alters the lipid content of cardiac myocytes [30]. 

Overall, previous findings, indicate that mice ApoA1−/−, with low levels of HDL presented modified properties of the L-type calcium channel (ICa-L), resulting in a decrease of upstroke velocity and increased duration of the action potential and QT interval duration, compared with wild type mice [30].

To date it is known that a variety of ion channels (Ca^2++^ sensitive, K^+^ channels, and Na^+^ and Ca^2+^ voltage-gated) are regulated by changes in the amount of membrane cholesterol and usually down-regulated by its increase [31].

Our findings indicate that the association between HDL cholesterol and the QTc interval was statistically significant only in women; a gender-difference that could be explained by the fact that electrophysiological properties of the ventricles are different between men and women [32,33]. 

Moreover, experimental findings showed sex differences in the ion channel ICa-L level expression in the left ventricle of the heart [34]. More precisely, in the animal model, ICa-L is more represented in male than female myocytes in the base of the heart but not in the apex, resulting in males only, in a baso-apical gradient of ICa-L concentration [34]. A different gene distribution in men and women of the ICa-L channels, which might also include a different susceptibility to HDL, was also demonstrated in the human heart [35,36].

We can thus speculate that the gender dependent HDL modulating effect on QT could be related to the specific ICa-L channel myocardial distribution pattern and that incremental changes in serum lipids could impact men and women in a different way, translating in a more pronounced HDL-C cardio protective effect in females [37]. 

Confirming this hypothesis, in a 26-year follow-up of the Framingham study, analyzing the risk for CHD in relation to the total cholesterol-to-HDL ratio for men and women, a high ratio was particularly important for women with a 3% risk reduction for each 1 mg/dL increase in HDL compared to a 2% only for men [38]. Furthermore, the antiarrhythmic effect of HDL was explored in the context of atrial fibrillation, demonstrating a significant gender difference, with a significant increase per 10-mg/dL of HDL decrease (HR 1.32 (1.66–1.05)) of AF risk in woman but not in men [39]. 

It is probable that sex hormones play a role in the observed gender association. Notably, an inverse relationship between progesterone levels and QT duration was demonstrated [40,41]. The proposed mechanism was a suppressive effect of sex hormones on ventricular ICa-L channels.

Our data indicate in women, a J-shaped association between HDL and the risk of QTc prolongation, suggesting a higher risk of QTc prolongation for the highest levels of HDL. We cannot provide a precise justification for this finding, but several potential reasons could explain the conundrum. We could hypothesize that this aspect might be linked to the well known non linear relationship between HDL-C and cardiovascular risk, leading to a paradoxical association between higher mortality and cardiovascular risk, and extremely high concentrations of HDL [42,43]. Furthermore, recent findings of a large population-based cohort study in Denmark, showed increasing CV mortality rates among European Caucasians with HDL levels over 1.9 mmol/L for men and over 2.4 mmol/L for women [44]. Results consistent with our findings, in which the spline regression analysis suggests a higher risk of QTc prolongation in women with HDL values above 2.5 mmol/L. It is however important to note that the confidence intervals related to the odds ratio estimates for QTc prolongation, shown in the spline regression analysis of Figure 3, mainly due to the reduced number of observations, significantly diverge at the extremes of HDL concentration. This fact could influence the reliability of the estimates and it is a well-known constraint of this kind of epidemiological studies [44]. Nevertheless, performing joint linear and logistic regression analyses, both suggesting a mutual relationship between HDL and QTc, we tried to overcome the limitation.

If the epidemiological association between very high levels of HDL and CV risk is well known, the biological linkage underlining this association represents, to date, an open issue. Genetic variants or unidentified acquired conditions, which could lead to the production of dysfunctional HDL, and to an acceleration of atherosclerotic processes have been evoked [45,46,47]. In this subset of subjects, HDL levels do not match HDL function, and the measurement of HDL concentration (matching in fact the cholesterol content of HDL particles only) does not appear to be a reliable predictor of HDL function [48]. However, considering the large and robust amount of results of previous studies, confirming the association between HDL levels and CV risk, HDL cholesterol value determinations remain a cornerstone in cardiovascular risk assessment.

Our study presents several strengths: It represents the largest population-based study to date aimed at exploring the topic, it is based on a comprehensive approach, and it takes into account a large number of covariates for the adjusted models. We also conducted an extensive analysis of the QTc interval using two different formulas, Bazett and Framingham, for the correction. The Framingham formula, rather than the Bazett’s, is based on empirical data from a large population sample and on linear regression functions and is recommended in recent guidelines [24]. However, for comparability with previous studies, we explored the association also for the widely used Bazett’s formula obtaining similar results. In our study we were able to adjust analyses for several potential key confounders, showing that the association in women also persists after adjustment. A remodeling effect of HDL with consequences on cardiac electrophysiology can be postulated.

Regardless of the epidemiological association found in our study, the important question, which arises, is: In which way the present findings can be translated in a clinical perspective? The clinical relevance of the results was not directly investigated, however some aspects have to be mentioned. Even if to date, LDL cholesterol represents the most efficient and moldable therapeutic target, in cardiovascular management, attempts to raise HDL have been included in risk management. Decreasing body weight (especially in obese patients) and increasing physical activity have been in fact found to improve HDL levels and function and triglyceride rich lipoprotein concentrations [49].

Our findings, even if far away from the evidence driving clinical practice, could help to further motivate attempts and targeted investigations aimed at raising HDL in obese patients, patients with type 2 diabetes, and patients affected by metabolic syndrome, which show a peculiar dyslipidemic feature, characterized by low levels of HDL [50]. An additional reason for which the cited sub-population, should represent a suitable target, is highlighted by previous findings demonstrating an increased risk of QTc prolongation in these classes of patients [51]. Moreover, going ahead in speculating on possible clinical implications, the QT interval prolongation risk in patient with low levels of HDL taking drugs interfering with cardiac repolarization, (e.g., antidepressant, antibiotics, etc.), should ideally be investigated. The same could be true for patients with electrolyte abnormalities increasing the risk of prolonged QTc including hypokalemia and hypomagnesemia. Last but not least, our findings could stimulate future research aimed to better understand the role of HDL cholesterol in cardiac repolarization.

Even if beyond the purposes of our study, our results could raise several questions in the public health context as well. Our data further underline the relevance of the HDL cholesterol measurement in estimating the global cardiovascular risk, as already stated and included in different algorithms [52,53]. The guidelines for the treatment of High Blood Cholesterol in Adults (Adult Treatment Panel III) of the National Cholesterol Education Program, clearly indicate low HDL cholesterol levels (defined as a concentration <40 mg/dL) as a therapeutic target and a cutoff for the classification of CV risk [54]. Epidemiological data and results of clinical trials clearly show how increasing HDL cholesterol levels can help in reducing the risk of coronary heart disease (an increase of 1 mg/dL corresponds to a CV events reduction of 2% in men and 3% in women [38]. Considering the beneficial effect of life style strategies impacting HDL, such as nutritional intervention, exercise, stress reduction, and tobacco and alcohol cessation, a benefit in terms of public health in targeted attempts was postulated [55].

Our results however indicate an epidemiological association that does not necessarily imply causality and/or potential intervention targets in the wide spectrum of arrhythmic and atherosclerotic cardiovascular diseases. Replication studies, translational research and clinical trials are needed to better understand if HDL has a role on cardiac repolarization.

## 5. Study Limitations

The main limitation of the study was the cross-sectional design, whereby even if an epidemiological association between HDL cholesterol and QTc was found; it does not prove causality of the relationship. Moreover, the observational nature of the study did not prove the temporality of the HDL cholesterol and QTc association; the direction of causality of the relationship not being inferable. We have to highlight the need for replication studies, in other populations and of cohort prospective studies aimed at confirming the association between HDL and QTc and at establishing the temporality and causality of this relationship.

A further limitation was represented by the endpoint studied, which represents a surrogate clinical outcome only. This meaning that even if an association between HDL cholesterol and QTc was found, this does not automatically imply a similar association between HDL and hard clinical related outcomes like ventricular arrhythmias and sudden cardiac death.

Moreover the observational character of our study made it difficult to provide deeper insights into underlying (patho)-physiological mechanisms, the observational nature of the data exposed them to residual unmeasured confounders and participants were predominantly Caucasian, limiting inference to other ethnic groups. Lastly, we acknowledged that the QT interval, known to be dynamic, was only measured from a baseline resting ECG in our study.

## 6. Conclusions

Findings of the present study indicated an epidemiological J-shaped association between QTc duration and HDL cholesterol. Investigations in other populations and in cohort prospective studies are needed to better define the nature of the association and to explore the causality of the relationship. The potential pleiotropic antiarrhythmic effects of HDL cholesterol on cardiac repolarization still remains an open field of investigation and matter for speculation.

## Figures and Tables

**Table 1:** Characteristics of the study population.

Clinical Characteristics	Number or Median	(25th–75th Percentile) or %
Age, years	51	42–59
Gender, Females	619	57.1 (%)
Weight, Kg	70	59–81
Height, cm	168	162–176
BMI, Kg/m^2^	24	22–27
Waist/Hip, cm	0.91	0.86–0.95
Systolic Blood Pressure in-office, mmHg	129	119–140
Diastolic Blood Pressure in-office, mmHg	80	74–88
**Medical History**		
Smoking	217	20 (%)
Family history of CVD	265	24 (%)
Hypercholesterolemia	128	12 (%)
Hypertension	144	13 (%)
Diabetes mellitus	18	2 (%)
Statin therapy	130	13 (%)
**Laboratory Characteristics**		
Total Cholesterol, mmol/L	5.3	4.6–6.0
LDL, mmol/L	3.5	2.9–4.2
HDL, mmol/L	1.6	1.3–1.9
HDL, <25th percentile (<1.3 mmol/L)	339	31 (%)
HDL, 25th to 50th percentile (1.4–1.6 mmol/L)	304	28 (%)
HDL, 50th to 75th percentile (1.7–1.9 mmol/L)	231	21 (%)
HDL, >75th percentile (>2.0 mmol/L)	209	19 (%)
Triglycerides, mmol/L	0.90	0.7–1.3
Magnesium, mmol/L	0.83	0.83–0.87
Calcium, mmol/L	1.21	1.18–1.22
Potassium, mmol/24 h	35	26–49
Sodium, mmol/L/24 h	165	115–233
Albumin, mg/24 h	25.2	21.1-28.9
Creatinin, μmol/L	16.5	11.2–23.5
Glomerular filtration rate, mL/min/1.73 m² (CKD-EPI creatinine equation)	96.1	85.7–105.8
Blood Urea Nitrogen, mmol/L	354	278–445
Cystatin, mg/L	0.80	0.73–0.89
Hemoglobin A1c, (%)	5.3	5.1–5.5
Glycemia, mmol/L	5.8	5.5–6.2
**Ambulatory Blood Pressure Monitoring**		
Systolic Blood Pressure, mmHg/24 h	117	111–126
Diastolic Blood Pressure, mmHg/24 h	73	68–80
Heart Rate, beats/min/24 h	67	65–75
Pulse Wave Velocity, m/sec/24/h	6.9	5.93–8.0
**ECG Variables**		
Heart Rate, beats/min	66	59–73
PR Interval, ms	152	140–168
QRS Interval, ms	86	80–94
QT, ms	406	390–426
QTc_Fram,_ ms	420	408–432
QTc_Bazz_, ms	427	413–441

Values are *n* (%) or median (25th–75th percentile). ECG: Electrocardiogram; QTc_Fram_: QT interval corrected for heart rate using the Framingham formula; QTc_Bazz_: QT interval corrected for heart rate using the Bazett formula.

**Table 2:** Linear regression exploring the correlation between high-density lipoprotein cholesterol (HDL) and QT interval corrected for heart rate (QTc_Fram_ and QTc_Bazz_) in women and men.

	Women						Men					
	Model 1 Unadjusted			Model 2 Adjusted			Model 1 Unadjusted			Model 2 Adjusted		
QTc_Bazz_, ms	β-coef	SE	*p*-value	β-coef	SE	*p*-value	β-coef	SE	*p*-value	β-coef	SE	*p*-value
**HDL, <25th percentile** **(≤1.3 mmol/L)**	**Reference**						**Reference**					
**HDL, 25th to 50th percentile ** **(1.4–1.6 mmol/L)**	–11.306	4.625	0.016	–11.697	4.333	<0.001	–2.047	4.297	0.635	1.197	4.025	0.625
**HDL, 50th to 75th percentile ** **(1.7–1.9 mmol/L)**	–12.347	4.875	0.012	–11.786	4.719	0.014	–8.450	5.098	0.100	–1.133	5.039	0.792
**HDL, >75th percentile** **(≥2.0 mmol/L)**	–5.047	4.658	0.280	–4.937	4.626	0.288	3.162	7.343	0.667	10.447	6.805	0.128
**QTc_Fram,_ ms**												
**HDL, <25th percentile** **(≤1.3 mmol/L)**	**Reference**						**Reference**					
**HDL, 25th to 50th percentile** **(1.4–1.6 mmol/L)**	–10.186	4.016	0.012	–10.908	4.151	0.010	–0.440	3.759	0.907	2.007	3.967	0.614
**HDL, 50th to 75th percentile** **(1.7–1.9 mmol/L)**	–12.048	4.233	<0.001	–11.002	4.521	0.016	–3.423	4.459	0.444	–1.298	4.966	0.794
**HDL, > 75 th percentile** **(≥2.0 mmol/L)**	–5.985	4.045	0.141	–4.368	4.432	0.326	14.072	6.423	0.030	10.644	6.076	0.116

## Supplementary Materials

The following are available online at https://www.mdpi.com/2077-0383/8/10/1527/s1.

## References

[1] Imaizumi S., Miura S., Nakamura K., Kiya Y., Uehara Y., Zhang B., Matsuo Y., Urata H., Ideishi M., Rye K.A. (2008). Antiarrhythmogenic effect of reconstituted high-density lipoprotein against ischemia/reperfusion in rats. J. Am. Coll. Cardiol..

[2] Mochizuki S., Okumura M., Tanaka F., Sato T., Kagami A., Tada N., Nagano M. (1991). Ischemia-reperfusion arrhythmias and lipids: Effect of human high- and low-density lipoproteins on reperfusion arrhythmias. Cardiovasc. Drugs Ther..

[3] Kunutsor S.K., Zaccardi F., Karppi J., Kurl S., Laukkanen J.A. (2017). Is High Serum LDL/HDL Cholesterol Ratio an Emerging Risk Factor for Sudden Cardiac Death? Findings from the KIHD Study. J. Atheroscler. Thromb..

[4] Wannamethee G., Shaper A.G., Macfarlane P.W., Walker M. (1995). Risk factors for sudden cardiac death in middle-aged British men. Circulation.

[5] Kirchhof P., Fabritz L. (2011). High-density lipoprotein shortens the ventricular action potential. A novel explanation for how statins prevent sudden arrhythmic death?. J. Am. Coll. Cardiol..

[6] Liu Y.B., Wu C.C., Lee C.M., Chen W.J., Wang T.D., Chen P.S., Lee Y.T. (2006). Dyslipidemia is associated with ventricular tachyarrhythmia in patients with acute ST-segment elevation myocardial infarction. J. Formos. Med. Assoc..

[7] Boudi F.B., Kalayeh N., Movahed M.R. (2018). High-Density Lipoprotein Cholesterol (HDL-C) Levels Independently Correlates with Cardiac Arrhythmias and Atrial Fibrillation. J. Intensive Care Med..

[8] Li Z.Z., Du X., Guo X.Y., Tang R.B., Jiang C., Liu N., Chang S.S., Yu R.H., Long D.Y., Bai R. (2018). Association Between Blood Lipid Profiles and Atrial Fibrillation: A Case-Control Study. Med. Sci. Monit..

[9] Yao H., Jiang L., Lin X., Liang Z.G. (2016). Fluvastatin combined with benazepril may contribute to the favorable prognosis of patients with atrial fibrillation. Biomed. Pharmacother..

[10] Annoura M., Ogawa M., Kumagai K., Zhang B., Saku K., Arakawa K. (1999). Cholesterol paradox in patients with paroxysmal atrial fibrillation. Cardiology.

[11] Hayashi M., Shimizu W., Albert C.M. (2015). The spectrum of epidemiology underlying sudden cardiac death. Circ. Res..

[12] Straus S.M., Kors J.A., De Bruin M.L., van der Hooft C.S., Hofman A., Heeringa J., Deckers J.W., Kingma J.H., Sturkenboom M.C., Stricker B.H. (2006). Prolonged QTc interval and risk of sudden cardiac death in a population of older adults. J. Am. Coll. Cardiol..

[13] Roden D.M. (2008). Keep the QT interval: It is a reliable predictor of ventricular arrhythmias. Heart Rhythm.

[14] Den Ruijter H.M., Franssen R., Verkerk A.O., van Wijk D.F., Vaessen S.F., Holleboom A.G., Levels J.H., Opthof T., Sungnoon R., Stroes E.S. (2011). Reconstituted high-density lipoprotein shortens cardiac repolarization. J. Am. Coll. Cardiol..

[15] O’Neal W.T., Singleton M.J., Roberts J.D., Tereshchenko L.G., Sotoodehnia N., Chen L.Y., Marcus G.M., Soliman E.Z. (2017). Association Between QT-Interval Components and Sudden Cardiac Death: The ARIC Study (Atherosclerosis Risk in Communities). Circ. Arrhythm. Electrophysiol..

[16] Williams E.S., Thomas K.L., Broderick S., Shaw L.K., Velazquez E.J., Al-Khatib S.M., Daubert J.P. (2012). Race and gender variation in the QT interval and its association with mortality in patients with coronary artery disease: Results from the Duke Databank for Cardiovascular Disease (DDCD). Am. Heart J..

[17] Beinart R., Zhang Y., Lima J.A., Bluemke D.A., Soliman E.Z., Heckbert S.R., Post W.S., Guallar E., Nazarian S. (2014). The QT interval is associated with incident cardiovascular events: The MESA study. J. Am. Coll. Cardiol..

[18] Korantzopoulos P., Liberopoulos E., Barkas F., Kei A., Goudevenos J.A., Elisaf M. (2014). No association between high-density lipoprotein levels and ventricular repolarization indexes in subjects with primary hypercholesterolemia. Scand. J. Clin. Lab. Investig..

[19] Vandenbroucke J.P., von Elm E., Altman D.G., Gøtzsche P.C., Mulrow C.D., Pocock S.J., Poole C., Schlesselman J.J., Egger M. (2007). Strengthening the Reporting of Observational Studies in Epidemiology (STROBE): Explanation and elaboration. PLoS Med..

[20] TRUE Consortium (2017). Recommended Standards for Assessing Blood Pressure in Human Research where Blood Pressure or Hypertension Is a Major Focus. Kidney Int. Rep..

[21] Salvadé I., Schätti-Stählin S., Violetti E., Schönholzer C., Cereghetti C., Zwahlen H., Berwert L., Burnier M., Gabutti L. (2015). A prospective observational study comparing a non-operator dependent automatic PWV analyser to pulse pressure, in assessing arterial stiffness in hemodialysis. BMC Nephrol..

[22] Milan A., Zocaro G., Leone D., Tosello F., Buraioli I., Schiavone D., Veglio F. (2019). Current assessment of pulse wave velocity: Comprehensive review of validation studies. J. Hypertens..

[23] Levey A.S., Stevens L.A., Schmid C.H., Zhang Y.L., Castro A.F., Feldman H.I., Kusek J.W., Eggers P., Van Lente F., Greene T. (2009). A new equation to estimate glomerular filtration rate. Ann. Intern. Med..

[24] Rautaharju P.M., Surawicz B., Gettes L.S. (2009). AHA/ACCF/HRS Recommendations for the Standardization and Interpretation of the Electrocardiogram Part IV: The ST Segment, T and U Waves, and the QT Interval A Scientific Statement from the American Heart Association Electrocardiography and Arrhythmias Committee, Council on Clinical Cardiology; the American College of Cardiology Foundation; and the Heart Rhythm Society Endorsed by the International Society for Computerized Electrocardiology. J. Am. Coll. Cardiol..

[25] Dettori J.R., Norvell D.C., Chapman J.R. (2018). The Sin of Missing Data: Is All Forgiven by Way of Imputation?. Glob. Spine J..

[26] Maguy A., Hebert T.E., Nattel S. (2006). Involvement of lipid rafts and caveolae in cardiac ion channel function. Cardiovasc. Res..

[27] Abi-Char J., Maguy A., Coulombe A., Balse E., Ratajczak P., Samuel J.L., Nattel S., Hatem S.N. (2007). Membrane cholesterol modulates Kv1.5 potassium channel distribution and function in rat cardiomyocytes. J. Physiol..

[28] Levitan I., Singh D.K., Rosenhouse-Dantsker A. (2014). Cholesterol binding to ion channels. Front. Physiol..

[29] Balijepalli R.C., Kamp T.J. (2008). Caveolae, ion channels and cardiac arrhythmias. Prog. Biophys. Mol. Biol..

[30] Baartscheer A., Schumacher C.A., Wekker V., Verkerk A.O., Veldkamp M.W., van Oort R.J., Elzenaar I., Ottenhoff R., van Roomen C., Aerts H. (2015). Dyscholesterolemia Protects Against Ischemia-Induced Ventricular Arrhythmias. Circ. Arrhythm. Electrophysiol..

[31] Levitan I., Fang Y., Rosenhouse-Dantsker A., Romanenko V. (2010). Cholesterol and ion channels. Subcell. Biochem..

[32] Trépanier-Boulay V., St-Michel C., Tremblay A., Fiset C. (2001). Gender-based differences in cardiac repolarization in mouse ventricle. Circ. Res..

[33] Yarnoz M.J., Curtis A.B. (2008). More reasons why men and women are not the same (gender differences in electrophysiology and arrhythmias). Am. J. Cardiol..

[34] Sims C., Reisenweber S., Viswanathan P.C., Choi B.R., Walker W.H., Salama G. (2008). Sex, age, and regional differences in L type calcium current are important determinants of arrhythmia phenotype in rabbit hearts with drug-induced long QT type 2. Circ. Res..

[35] Chu S.H., Sutherland K., Beck J., Kowalski J., Goldspink P., Schwertz D. (2005). Sex differences in expression of calcium-handling proteins and beta-adrenergic receptors in rat heart ventricle. Life Sci..

[36] Furukawa T., Kurokawa J. (2007). Regulation of cardiac ion channels via non-genomic action of sex steroid hormones: Implication for the gender difference in cardiac arrhythmias. Pharmacol. Ther..

[37] Legato M.J. (2000). Dyslipidaemia, gender, and the role of high density lipoprotein cholesterol: Implications for therapy. Am. J. Cardiol..

[38] Gordon D.J., Probstfield J.L., Garrison R.J., Neaton J.D., Castelli W.P., Knoke J.D., Jacobs D.R., Bangdiwala S., Tyroler H.A. (1989). High-density lipoprotein cholesterol and cardiovascular disease: Four prospective American studies. Circulation.

[39] Watanabe H., Tanabe N., Yagihara N., Watanabe T., Aizawa Y., Kodama M. (2011). Association between lipid profile and risk of atrial fibrillation. Circ. J..

[40] Nakagawa M., Ooie T., Ou B., Ichinose M., Takahashi N., Hara M., Yonemochi H., Saikawa T. (2005). Gender differences in autonomic modulation of ventricular repolarization in humans. J. Cardiovasc. Electrophysiol..

[41] Zhang Y., Ouyang P., Post W.S., Dalal D., Vaidya D., Blasco-Colmenares E., Soliman E.Z., Tomaselli G.F., Guallar E. (2011). Sex-steroid hormones and electrocardiographic QT-interval duration: Findings from the Third National Health and Nutrition Examination Survey and the Multi-Ethnic Study of Atherosclerosis. Am. J. Epidemiol..

[42] Wilkins J.T., Ning H., Stone N.J., Criqui M.H., Zhao L., Greenland P., Lloyd-Jones D.M. (2014). Coronary heart disease risks associated with high levels of HDL cholesterol. J. Am. Heart Assoc..

[43] Van der Steeg W.A., Holme I., Boekholdt S.M., Larsen M.L., Lindahl C., Stroes E.S.G., Tikkanen M.J., Wareham N.J., Faergeman O., Olsson A.G. (2008). High-density lipoprotein cholesterol, high-density lipoprotein particle size, and apolipoprotein A-I: Significance for cardiovascular risk: The IDEAL and EPIC-Norfolk studies. J. Am. Coll. Cardiol..

[44] Madsen C.M., Varbo A., Nordestgaard B.G. (2017). Extreme high high-density lipoprotein cholesterol is paradoxically associated with high mortality in men and women: Two prospective cohort studies. Eur. Heart J..

[45] Voight B.F., Peloso G.M., Orho-Melander M., Frikke-Schmidt R., Barbalic M., Jensen M.K. (2012). Plasma HDL cholesterol and risk of myocardial infarction: A mendelian randomisation study. Lancet.

[46] Zanoni P., Khetarpal S.A., Larach D.B., Hancock-Cerutti W.F., Millar J.S., Cuchel M., DerOhannessian S., Kontush A., Surendran P., Saleheen D. (2016). Rare variant in scavenger receptor BI raises HDL cholesterol and increases risk of coronary heart disease. Science.

[47] Rosenson R.S., Brewer H.B., Ansell B.J., Barter P., Chapman M.J., Heinecke J.W., Kontush A., Tall A.R., Webb N.R. (2016). Dysfunctional HDL and atherosclerotic cardiovascular disease. Nat. Rev. Cardiol..

[48] Arsenault B.J., Despre’s J.P. (2012). HDL cholesterol is not HDL—Don’t judge the book by its cover. Nat. Rev. Cardiol..

[49] Adams V., Besler C., Fischer T., Riwanto M., Noack F., Höllriegel R., Oberbach A., Jehmlich N., Völker U., Winzer E.B. (2013). Exercise training in patients with chronic heart failure promotes restoration of high-density lipoprotein functional properties. Circ. Res..

[50] Wu L., Parhofer K.G. (2014). Diabetic dyslipidemia. Metabolism.

[51] Arslan E., Yiğiner O., Yavaşoğlu Ozçelik F., Kardeşoğlu E., Nalbant S. (2010). Effect of uncomplicated obesity on QT interval in young men. Pol. Arch. Med. Wewn..

[52] Reiner Z., Catapano A.L., De Backer G., Graham I., Taskinen M.-R., Wiklund O., Agewall S., Alegria E., Chapman M.J., Durrington P. (2011). ESC/EAS Guidelines for the management of dyslipidaemias: The Task Force for the management of dyslipidaemias of the European Society of Cardiology (ESC) and the European Atherosclerosis Society (EAS). Eur. Heart J..

[53] Hamer M., O’Donovan G., Stamatakis E. (2018). High-Density Lipoprotein Cholesterol and Mortality: Too Much of a Good Thing?. Arterioscler. Thromb. Vasc. Biol..

[54] (2001). Executive summary of the third report of the National Cholesterol Education Program (NCEP) Expert Panel on Detection, Evaluation, and Treatment of High Blood Cholesterol in Adults (Adult Treatment Panel III). JAMA.

[55] Farrer S. (2018). Beyond Statins: Emerging Evidence for HDL-Increasing Therapies and Diet in Treating Cardiovascular Disease. Adv. Prev. Med..

