# Supplementary material for: Association Between HDL Cholesterol and QTc Interval: A Population-Based Epidemiological Study
Source: J Clin Med. 2019 Sep 23;8(10):1527. doi: 10.3390/jcm8101527 (PMC6832837; doi:10.3390/jcm8101527)
Supplement: Supplementary file 1 [file jcm-08-01527-s001.pdf]

Supplementary Table 1

	Women							Men				
	QTcBaz Normal	QTcBaz Prolonged	p-value	QTc Fram Normal	QTc Fram Prolonged	p-value	QTcBaz Normal	QTcBaz Prolonged	p-value	QTc Normal	QTc Prolonged	p-value
Age, y	51 (42-59)	51 (44-69)	0.206	51 (42-58)	52(44-65)	0.029	50 (42-58)	54 (44- 62)	0.1453	50 (42-58)	54 (44-63)	0.039
Weight, Kg	61 (55-70)	64 (57-72)	0.216	61 (55-70)	62 (55-69)	0.536	79 (73-88)	80 (72-91)	0.1581	80 (73-88)	77 (72-89)	0.963
Height, cm	163 (159-168)	163 (160-167)	0.804	163 (159-169)	163 (159-168)	0.752	176 (172-181)	176 (171-182)	0.649	176 (172-181)	176 (171-180)	0.835
BMI, Kg/m^2	23.1 (22-26)	23.4 (21.1-27.4)	0.181	23.1 (21-26)	23 (21-26)	0.0.54	25.5 (23.5-28)	25.6 (23.4-29.4)	0.106	25.5(23.5-27.8)	25.3(23.4-28.7)	0.894
Waist/Hip, cm	0.87 (0.83-0.91)	0.89 (0.85-0.93)	<0.001	0.88 (0.83-0.91)	0.87 (0.84-0.92)	0.095	0.95 (0.91-0.98)	0.95 (0.93- 0.99)	0.571	0.95 (0.91-0.98)	0.94 (0.90-0.99)	0.121
SBP, mmHg	125 (117-136)	132 (124-154)	<0.001	126 (117-136)	131 (122-146)	0.05	136 (127-146)	142 (129 -158)	<0.001	137 (127-147)	134 (127-146)	0.865
DBP, mmHg	78 (71-85)	84 (76-98)	<0.001	78 (71-86)	81 (73-90)	0.05	84 (78-92)	89 (78-98)	0.014	84 (78-93)	82 (75-90)	0.108
Smoking	20.1	15.6	0.426	20.7	14.9	0.199	20.8	12. 8	0.225	21.3	12.8	0.171
Family history of CVD	24.7	37.8	0.05	23.6	35.6	0.013	22.4	30.8	0.232	22.2	31.9	0.13
Hypercholesterolemia	11.3	8.9	0.617	11.8	13.8	0.583	12.9	13.2	0.974	16	23.4	0.196
Hypertension	10.4	17.8	0.049	12.5	24.1	0.002	16.5	18.4	0.762	12.8	14.9	0.691
Diabetes	1.2	6.7	0.001	1.3	4.9	0.027	1.7	2.6	0.657	1.7	2.1	0.830
Statin therapy	11.9	6.7	0.239	12.1	12.6	0.883	12.9	13.2	0.974	12.8	14.9	0.691
Total Cholesterol, mmol/L	5.3 (4.6-6.0)	5.3(4.7-5.9)	0.966	5.2 (4.6-6.0)	5.5 (4.8-5.9)	0.853	5.3 (4.6-6.0)	5.5 (4.9- 6)	0.567	5.3 (4.6-6.1)	5.3 (4.7 -5.9)	0.225
LDL, mmol/L	3.4 (2.8-4.1)	3.5 (2.9-3.9)	0.658	3.4 (2.8-4.1)	3.6 (2.9-4)	0.641	3.7 (3 -4.3)	3.9 (3.1-4.3)	0.997	3.7 (3.05-4.4)	3.8 (2.9-4.2)	0.256
HDL, mmol/L	1.7 (1.5-2)	1.5 (1.2-2.2)	0.507	1.7 (1.5-2.0)	1.8 (1.4-2.1)	0.578	1.3 (1.1-1.6)	1.4 (1.2 -1.7)	0.197	1.3(1.1-1.6)	1.5 (1.2-1.8)	0.002
HDL, <25th percentile	14.7	33.3	<0.001	31.7	27.6	0.433	51.2	48.72	0.705	53.4	36.2	0.025
HDL, 25th to 50th percentile	30.8	17.8	0.07	28.6	21.2	0.177	25.82	25.64	0.980	25.5	27.7	0.746
HDL, 50th to 75th percentile	26.3	13.3	0.06	21.7	16.1	0.222	15.26	20.5	0.388	15	25.5	0.06
HDL, > 75th percentile	28.2	35.6	0.294	18	34.5	<0.001	7.1	5.1	0.651	6.5	10.6	0.288
Triglycerides, mmol/L	0.80 (0.60-1.1)	0.90 (0.70-1.40)	0.042	0.8 (0.6-1.1)	0.9 (0.7.-1.4)	0.126	1 (0.8-1.6)	1 (0.8-1.4)	0.018	1.1 (0.8-1.6)	0.9 (0.6-1.2)	0.144
Magnesium, mmol/L	0.83 (0.79-0.87)	0.82(0.77-0.86)	0.764	0.73 (0.79-0.87)	0.83 (0.78-0.87)	0.7286	0.84 (0.81-0.88)	0.87 (0.79-0.88)	0.799	0.84 (0.81-0.88)	0.85 (0.79-0.88)	0.487
Calcium, mmol/L	1.22 (1.20-1.24)	1.21(1.17-1.23)	0.013	1.22 (1.2-1.24)	1.22 (1.18-1.24)	0.223	1.22 (1.21.25)	1.2 (1.2-1.24)	0.879	1.2 (1.2-1.25)	1.23 (1.21-1.2)5	0.5780
Potassium (urinary), mmol/24h	54 (42-69)	54 (42-66)	0.412	55 (42-69)	50.5 (40-67)	0.451	67 (55-83)	70 (55-81)	0.877	67 (54 -82)	72 (59-83)	0.360
Sodium (urinary), mmol/24h	141 (99-198)	148 (97-187)	0.381	142 (101-197.5)	126.5(90.5-189)	0.195	204 (145-278)	181 (125-254)	0.110	208.5 (147-280)	155 (124-243)	0.032
Creatinine, µmol/L	66(59-74)	63(56-74)	0.746	66 (59-74)	68 (61-75)	0.084	84 (76-94)	83 (73- 91)	0.3261	84 (76-94)	84 (74 -92)	0.796
GFR, mL/min/1.73m²	97.4 (88-110.5)	98.7(91.3-108.9)	0.348	97.4 (88-110.5)	98(86.9-105.8)	0.236	92.5 (83.9-101.4)	93.7 (81.2-107.3)	0.507	92.5 (83.9-101)	91.3 (80.1-105.8)	0.826
Urea (urinary), mmol/24h	308 (254-382)	269(228-354)	0.075	307.5(253-379.1)	295.6 (228.3-388)	0.419	439.5 (350-525)	453.3 (356.6-503.3)	0.693	440 (351-523)	439.5 (356.6-542.6)	0.380
Cystatin C, mg/L	0.79(0.70-0.87)	0.78(0.71-0.84)	0.143	0.79(0.70-0.87)	0.79 (0.73-0.88)	0.219	0.83 (0.76-0.91)	0 .82 (0.72-0 .94)	0.684	0.83 (0.76-0.91)	0.84 (0.73-0.95)	0.793
Hemoglobin A1c, (%)	5.3 (5-5.5)	5.3 (5.1-5.7)	<0.001	5.3 (5.1-5.6)	5.3 (5.1-5.6)	0.2763	5.3 (5.1-5.5)	5.4 (5.1-5.6)	0.098	5.3 (5.1-5.5)	5.3 (5.1 -5.5)	0.422
Glycemia, mmol/L	5.8 (5.4-6.2)	5.8 (5.5-6.5)	0.004	5.8 (5.2-6.2)	5.8 (5.6-6.3)	0.272	5.8 (5.5-6.2)	6 (5.5 -6.3)	0.863	5.8 (5.5-6.2)	5.8 (5.5 -6.2)	0.887
SBP, mmHg (24h)	114(108-122)	122 (108-133)	<0.001	114(108-122)	117 (106-124)	0.3378	121 (115- 129)	120 (115-133)	0.439	121 (115-129)	124 (114-129)	0.324
DBP, mmHg (24h)	71 (66-76)	74 (67-83)	<0.001	71 (66-76)	71 (66-76)	0.372	78 (72-83)	77 (71-82.5)	0.595	78 (72-83)	78 (71-83)	0.867

HR, beats/min (24h)	71 (66-76)	73.5 (68-79)	0.017	71 (66-76.5)	67 (61-74)	<0.001	68 (62-74)	71(64.5-77.5)	0.093	69 (63-75)	64 (57- 71)	0.0001
PWV, m/sec (24h)	6.8 (5.9-7.9)	7.1 (5.9-9-3)	0.032	6.8 (5.9-7.9)	7. (5.9-9.3)	0.018	7 (6.1-7.9)	7.5 (6.3-8.6)	0.083	7.0 (6.1-7.8)	7.4 (6.3-9.2)	0.023
HR, beats/min	66 (60-73)	75 (70-82)	<0.001	67 (61-74)	65 (57-72)-	0.027	63 (57-70)	74 (67-79)	<0.001	64 (57 -71)	61 (55-69)	0.013
PR, ms	150 (136-164)	146 (132-158)	0.478	150(136-164)	152(136-164)	0.575	156 (144-174)	150 (138- 162)	0.273	156 (142-172)	152 (144-182)	0.811
QRS, ms	82 (78-88)	88 (90-92)	0.015	82(78-88)	86 (80-90)	0.016	92 (84-100)	96 (92-106)	0.002	92 (84-100)	98 (90-108)	<0.001
QT, ms	407 (390-424)	18 (400-436)	0.047	404 (390-421)	442 (422-458)	<0.001	404 (386-424)	422 (396 -430)	0.022	400 (386-420)	446 (430-462)	<0.001
